# The Role of Visual-Spatial Abilities in Dyslexia: Age Differences in Children’s Reading?

**DOI:** 10.3389/fpsyg.2016.01997

**Published:** 2016-12-21

**Authors:** Giulia Giovagnoli, Stefano Vicari, Serena Tomassetti, Deny Menghini

**Affiliations:** ^1^Department of Neuroscience, Bambino Gesù Children’s Hospital, IRCCSRome, Italy; ^2^Department of Human Studies, LUMSA UniversityRome, Italy

**Keywords:** learning disabilities, reading deficits, visual-spatial deficits, developmental disabilities

## Abstract

Reading is a highly complex process in which integrative neurocognitive functions are required. Visual-spatial abilities play a pivotal role because of the multi-faceted visual sensory processing involved in reading. Several studies show that children with developmental dyslexia (DD) fail to develop effective visual strategies and that some reading difficulties are linked to visual-spatial deficits. However, the relationship between visual-spatial skills and reading abilities is still a controversial issue. Crucially, the role that age plays has not been investigated in depth in this population, and it is still not clear if visual-spatial abilities differ across educational stages in DD. The aim of the present study was to investigate visual-spatial abilities in children with DD and in age-matched normal readers (NR) according to different educational stages: in children attending primary school and in children and adolescents attending secondary school. Moreover, in order to verify whether visual-spatial measures could predict reading performance, a regression analysis has been performed in younger and older children. The results showed that younger children with DD performed significantly worse than NR in a mental rotation task, a more-local visual-spatial task, a more-global visual-perceptual task and a visual-motor integration task. However, older children with DD showed deficits in the more-global visual-perceptual task, in a mental rotation task and in a visual attention task. In younger children, the regression analysis documented that reading abilities are predicted by the visual-motor integration task, while in older children only the more-global visual-perceptual task predicted reading performances. Present findings showed that visual-spatial deficits in children with DD were age-dependent and that visual-spatial abilities engaged in reading varied across different educational stages. In order to better understand their potential role in affecting reading, a comprehensive description and a multi-componential evaluation of visual-spatial abilities is needed with children with DD.

## Introduction

Developmental dyslexia (DD) is a specific learning disorder characterized by persistent difficulties in learning how to read accurately, fluently, and in reading comprehension caused by multiple genetic and environmental risk factors, as well as their interplay (Peterson and Pennington, 2015). The reading deficit should be sufficiently severe as to interfere with academic and occupational performances or with activities of daily living and it cannot be strictly due to intellectual disabilities, sensory disorders or inadequate educational instruction for it to be considered DD (American Psychiatric Association [APA], 2013). The ability to read entails the ability to transform written symbols, namely letters, into their corresponding sound and, then, to integrate these sounds onto one single word.

Developmental dyslexia is commonly described as a language-based disorder, in which the phonological domain is often compromised (Swan and Goswami, 1997; Snowling, 2000; Ramus et al., 2003; Shaywitz and Shaywitz, 2005; for a review Peterson and Pennington, 2012). However, several studies demonstrated that reading is a complex cognitive process, in which not only phonological skills, but also auditory sensory processes, memory abilities, attention processes, automatization and visual-spatial skills are involved (Nicolson and Fawcett, 1990; Pennington, 2006; Menghini et al., 2010b).

More specifically, visual-spatial processes have been documented to play a crucial role in reading and a number of studies reported a relationship between visual-spatial deficits and DD (Felmingham and Jakobson, 1995; Talcott et al., 1998, 2000; Vidyasagar and Pammer, 2010; Stein, 2014; Gori and Facoetti, 2015). However, contrasting results have been found in investigating visual-spatial abilities in DD. Behavioral studies demonstrated visual-spatial deficits in individuals with DD as they were shown to be impaired in different motion perception tasks (Menghini et al., 2010b; Boets et al., 2011; Gori et al., 2014, 2015), visual recognition tasks (Geiger et al., 2008) or in mental rotation tasks (Rüsseler et al., 2005). Consistently, a study by Winner et al. (2001) showed that adults and high school or undergraduate students with DD did not perform as successfully as control group in mental rotation, visual memory, spatial word problems and visual logical matrices regardless of attentional problems. Nevertheless, others studies failed to find similar deficits (Corballis et al., 1985; Del Giudice et al., 2000; Ramus et al., 2003; White et al., 2006). For example, in a study investigating the role of sensorimotor impairments in DD, no difference between motion coherence and visual stress has been found between aged-school children with DD and controls matched on gender, age and non-verbal IQ (White et al., 2006). A study carried out on high school students (von Károlyi et al., 2003) reported better performances in participants with DD with respect to normal readers (NR) in a specific visual-spatial task, such as rapid and accurate holistic inspection.

A crucial aspect for disentangling inconsistencies in the existing literature on visual-spatial abilities in DD could be the understanding of age-related changes in visual-spatial abilities and their relationship with reading. Indeed, the visual-spatial processing required changes for reading depends on the developmental reading phase (Hautus et al., 2003). Reading in children begins with the perception of letters and the analysis of their conventional phonetic value (Luria, 1966). To identify words, a child must first be able to recognize individual letters and perceive their ordering in space (Vernon, 1957). This is followed by a complex process: matching a symbol with a sound, putting them together and decoding symbols in order to construct or derive meaning. As reading skills develop, the analysis of individual letters is transformed into the direct recognition of words by sight (Luria, 1966; Ehri, 1987; Kuhn et al., 2006). Indeed, as children improve their reading skills, they start to recognize some words as a whole by their characteristic shape. In this expert stage of reading many processes are automatic, freeing up cognitive resources so that the readers possess semantic and syntactic information that enables them to form expectations about upcoming words in text and can reflect on meaning (Goodman, 1970). Fluent and automatic reading is thought to be achieved at the end of primary school (Schwanenflugel et al., 2006).

During primary school, a child will often devote a significant amount of mental capacity to the process of decoding, thus allowing the child to improve their decoding skills with the ultimate goal of developing the automatic process, as it is for most skilled readers with most text they encounter. As the skill of decoding improves and the more automatic it becomes, the more the child has mental capacity to devote to comprehension.

Many cognitive factors are involved in the process of learning how to read. During the earlier educational stages, children examine written words by a sequential decoding, in which attention to individual letter-sound associations, phonological awareness such as blending and segmentation, verbal working memory, and local visual analysis are specially required. In the following educational stages, with repeated exposure to words, the functioning of the phonological working memory becomes automated and children reach automatic recognition of the words, as a whole visual stimulus, and a strong activation of long-term memory stores is now required in order to support the reading (Nicolson and Fawcett, 1990; Pennington, 2006; Menghini et al., 2010a,b; Ruffino et al., 2014; Gori and Facoetti, 2015).

From a neurobiological point of view, different brain networks are involved in these different phases. According to Pugh et al. (2001), the dorsal brain circuits is at first engaged and performs the analytic processing necessary for learning to integrate orthographic with phonological and lexical–semantic features of words. Gradually, the ventral circuit attends to the reading process, in the word form system, underlying fluency in word recognition. A distinction between a ventral-lexical pathway and a dorsal-sublexical pathway has been confirmed also in several functional and structural studies (Pugh et al., 2000; Jobard et al., 2003; Borowsky et al., 2006, 2007; Steinbrink et al., 2008; Friederici et al., 2009).

Among the cognitive factors involved in reading, the present study aimed at better clarifying specific contribution of visual-spatial abilities in affecting reading skills of children with DD at different educational stages. In DD, reading deficits related to visual-spatial processing could be associated more in the first educational years to deficits in local analysis required for exploring letters and words, while a deficit in the global perceptual processing could affect more the following years when words should be analyzed for their global shapes. Difficulties in global perceptual processes could similarly affect the first educational stages since high-frequency words could be analyzed even in the first stages as a whole stimulus.

In order to explore the relationship between reading and visual-spatial abilities at different educational stages, participants were divided into two subgroups (younger and older) depending on whether they attended primary or secondary school. If the first educational stage involves both the analytic process for reading a new word and the more global process for recognizing already met words, while the next educational stage mainly needs more global process for reading already met words, then the contribution of visual-spatial deficits in reading of children with DD should vary across reading stages.

Our predictions were the following: first, we should observe that younger children with DD show poor performances in visual-spatial tasks elaborated by both dorsal and ventral pathway, while older children with DD show poor performances in more global visual perceptual tasks mainly processed by ventral pathway. Second, if different visual-spatial abilities are involved in reading process according to different educational stages, then reading performances should be predicted by distinct visual-spatial measures. Particularly, we should observe that reading in younger children is primarily predicted by both dorsal and ventral visual-spatial abilities while in older children by more ventral visual-spatial abilities.

## Materials and Methods

### Participants

Sixty right-handed children with DD (M/F = 33/27; mean age ± standard deviation = 11.4 ± 1.9, range = 8.4–17.6) and sixty-five NR (M/F = 37/28; mean age ± standard deviation = 11.9 ± 1.8, range = 8.1–15.7) participated in the study. Participants were recruited also for previous studies (Menghini et al., 2011; Varvara et al., 2014). The clinical diagnosis of DD was made on the basis of the DSM-IV criteria (American Psychiatric Association [APA], 2000) and national recommendations (Consensus Conference, 2007). Children with DD showed reading speed or accuracy level at least 2 standard deviations below the mean of their chronological age. Speed (in seconds) and errors were measured using the age-standardized “Battery for the evaluation of Developmental Dyslexia and Dysorthographia” (Sartori et al., 2007). NR performed within 1 standard deviation from the mean in reading tasks (speed and accuracy) and were matched to the children with DD for chronological age and cognitive abilities (see **Table 1**). Criteria for inclusion in the study were the following: a normal or corrected to normal visual acuity; and no other significant co-morbidity, like attention deficit or hyperactivity disorder (ADHD). The diagnosis of ADHD in the group with DD and in the control group was assessed on the basis of the ADHD rating scale for parents (Conners, 2000), as well as a clinical examination according to DSM-IV criteria. Afterward, we split participants into two subgroups based on different education stages and in accordance with previous studies using a similar cut-off (Biancarosa and Snow, 2006; Wexler et al., 2012).

**Table 1:** Chronological age, cognitive and reading measures of younger and older subgroups of children.

	DD	NR
	Mean (*SD*)	Mean (*SD*)
**Younger**		
Age (years)	9.78 (0.74)	10.09 (1.04)
CPM (percentile)	54.8 (26.4)	55.77 (25.4)
Word reading		
Speed (z-score)	–2.5 (2.76)	0.52 (0.72)
Accuracy (z-score)	–2.9 (2.11)	0.22 (0.45)
Word Inefficiency Index	237.5 (129.6)	92.63 (23.6)
Non-word reading		
Speed (z-score)	–1.73 (1.89)	0.11 (0.84)
Accuracy (z-score)	–2.47 (1.29)	0.18 (0.68)
Non-word Inefficiency Index	186.6 (89.8)	82.8 (25.33)
**Older**		
Age (years)	12.86 (1.36)	12.89 (1.27)
CPM (percentile)	71.59 (21.85)	67.28 (25.52)
Word reading		
Speed (z-score)	–3.90 (3.23)	0.43 (0.58)
Accuracy (z-score)	–4.13 (3.41)	0.04 (0.61)
Word Inefficiency Index	155.68 (73.69)	63.53 (15.79)
Non-word reading		
Speed (z-score)	–3.24 (2.06)	0.43 (0.84)
Accuracy (z-score)	–2.65 (2.17)	0.31 (0.56)
Non-word Inefficiency Index	136.9 (59.83)	50.74 (14.47)

*DD, Developmental Dyslexia; NR, Normal Readers; SD, Standard Deviation; CPM, Colored Progressive Matrices.*

The first subgroup included children with DD and NR attending the primary school, with a chronological age under 11 years old (younger) (respectively, *N* = 28 and *N* = 22). Since at the end of primary school fluent and automatic reading is generally expected to be achieved (Schwanenflugel et al., 2006), the second subgroups included children and adolescents with DD and NR in the secondary school, with a chronological age equal or above 11 years old (older) (respectively, *N* = 32 and *N* = 43).

Chronological age, cognitive abilities, and measures of reading abilities of subgroups are reported in **Table 1**. In both the younger and the older subgroups, participants with DD did not differ from NR in chronological age (younger DD vs. younger NR: *t*_(48)_ = -1.25, *p* = 0.22; older DD vs. older NR: *t*_(73)_ = -0.86, *p* = 0.93) and in cognitive abilities, as measured by Colored Progressive Matrices (CPM; Raven, 2010): younger DD vs. younger NR: *t*_(48)_ = 0.22, *p* = 0.98; older DD vs. older NR: *t*_(73)_ = 0.79, *p* = 0.43.

Children with DD were tested at the Children’s Hospital Bambino Gesù (Rome, Italy) while NR were evaluated individually in their school. Children were evaluated in two sessions on different days with each session lasting approximately 1 h and a half. Cognitive abilities and reading abilities were assessed in the first session while the remaining tasks were administered in the other sessions, in a pseudorandom way. A description of the tests is provided below.

### Ethics Statement

Before testing children, we obtained informed consent from all participants and their families, and the agreement by the local ethical committee (Protocol Number 486LB). Informed consent was given by the parents as well as the children. All children and families were informed through an information sheet, read to participant’s parents prior to asking their consent, with a copy handed to them to take home, and a separate sheet on which to record consent. Information about the project was explicitly written on the consent form, either in bullet points or as extended text. The name and signature of the person who took the participants through the consent procedure was recorded. The privacy of participants was guaranteed according to the data protection law. The study involved children with developmental reading disorder. Informed consent was given by parents and children. Children and families could withdraw their participation at any time in the study. Test administrators received intensive and specific training. Children were assessed in an encouraging and child-oriented manner.

### Design and Materials

Measures obtained by the participants in each task were transformed into z-scores to perform statistical analyses. The mean and the standard deviation were based on normative data of the tasks, except for SRT and STICK, in which the normative data were not available, and the mean and standard deviation of NR were used.

#### Cognitive Abilities

General intelligence was evaluated by CPM (Raven, 2010) and the scores were expressed in percentiles.

#### Reading Abilities

Speed and accuracy of reading were assessed using two subtests from the “Battery for the evaluation of Developmental Dyslexia and Dysorthography” (Sartori et al., 2007). In the first subtest, participants had to read aloud 4 lists of 28 concrete and abstract, high or low frequency words (length from 4 to 8 letters). In the second task children had to read three lists of 16 non-words (length from 5 to 9 letters). Speed (in seconds) and errors (each incorrect word or non-word was calculated as one error) were computed for each task and standardized using mean and standard deviation according to the class. An inefficiency reading index was devised to take into account both reading speed and accuracy and was separately computed for words and non-words. Each index was calculated as follows: the ratio between word or non-word reading speed (in seconds) and accuracy rate (number of correct words or non-words by the total number of words or non-words). Mean and standard deviation of word and non-word inefficiency index were included in **Table 1**.

#### Visual-Spatial Tasks

The visual-spatial perception abilities were evaluated using the subtests 2 and 4 from the Visual Perception Test (VPT; Hammill et al., 1994). VPT2, Visual Perception Test-subtest 2 is a visual-spatial ability task designed to investigate perceptual and discrimination capacities in the visual domain. Participants were asked to match one figure to another from a multiple-choice display consisting of an array of vertically arranged figures. In each of the 25 items, the wrong alternatives differed from the target due to minor changes in orientation or spatial relations between constitutive elements. VPT4, Visual Perception Test-subtest 4, measures the ability to distinguish an object from the background or from surrounding objects. Children were asked to identify the parts that one complex figure was made of. In more detail, participants were required to do a visual-object recognition, identifying two or more figures among other line drawings in a confusing context or within overlapping images.

Visual-spatial imagery and mental rotation abilities were evaluated using the Spatial Rotation Test (SRT; Vicari et al., 2006) and the Stick (STICK; Carlesimo et al., 2001). In each trial of SRT, children had to mentally rotate geometric figures to find the target among five alternatives drawn on a sheet of paper. In each trial of STICK, participants were presented with a line drawing of an L- or an S-shaped stick with a full or an empty circle at the two ends. They had to indicate which of four similarly shaped sticks, rotated from 45 to 270° on a horizontal plane, would match the stimulus stick after appropriate mental rotation based on the respective location of the full and the empty circles.

Selective visual-spatial attention was assessed using a subtest of the Test of Everyday Attention for Children (Map Mission, MAP; Manly et al., 2002). In this subtest, participants were presented with a color-printed A3-laminated city map, with eighty targets representing restaurants (i.e., small knife and fork symbols) randomly distributed across the map. Distracting symbols of the same size, such as supermarket trolleys, cups, or cars, were also present. Participants used a pen to circle as many targets as possible in 1 min. The performance score was calculated by the number of target symbols correctly marked by the participants.

Integration of visual input and motor output was measured using the Visual Motor Integration Test (VMI; Beery and Buktenica, 2000). Children were asked to copy geometric shapes on a sheet of paper. Overall scores were given by a qualitative evaluation of drawings, according to specific criteria.

For demonstrative purpose, means, standard deviation and raw score ranges for each visual-spatial measure were included in **Table 2**.

**Table 2:** Raw score range, mean, and standard deviation for each visual-spatial measure of younger and older subgroups of children.

	Raw score	DD	NR
	Range	Mean (*SD*)	Mean (*SD*)
**Younger**			
MAP	0–80	31.32 (8.27)	33.59 (7.55)
SRT	0–27	17.82 (5.71)	20.18 (3.59)
STICK	0–10	6.25 (1.97)	7.23 (2.36)
VPT2	0–25	20.11 (4.01)	22.36 (2.95)
VPT4	0–18	13.11 (2.42)	15 (1.38)
VMI	0–27	16.32 (2.54)	20.73 (3.61)
**Older**			
MAP	0–80	42.25 (9.48)	49.19 (9.77)
SRT	0–27	21.03 (3.14)	22.47 (2.59)
STICK	0–10	8 (1.95)	8.21 (2.01)
VPT2	0–25	22.22 (3.53)	23.33 (1.94)
VPT4	0–18	13.84 (3.31)	15.53 (2.04)
VMI	0–27	19.78 (3.05)	21.37 (2.96)

*DD, Developmental Dyslexia; SD, Standard Deviation; MAP, Map Mission; SRT, Spatial Rotation Test; STICK, Stick Test; VPT2, Visual Perception Test-subtest 2; VPT4, Visual Perception Test-subtest 4; VMI, Visual Motor Integration Task.*

### Statistical Analysis

Statistical analyses were performed using z-scores (see Design and Materials). In order to investigate if there were any differences in performing visual-spatial tasks according to educational stages, a MANOVA analysis was performed with Group (DD vs. NR) and Subgroup (younger vs. older) as between-subject factors and Task (STICK vs. MAP vs. SRT vs. VPT2 vs. VPT4 vs. VMI) as within-subject factors.

Pairwise comparisons between each group were analyzed through LSD *post hoc* tests.

To determine whether reading abilities were predicted by visual-spatial measures, a stepwise regression analysis in each subgroup (younger and older), with children with DD and NR as a whole group, was performed.

Two different regression analyses were computed with the inefficiency reading index (for words and non-words, separately) as dependent variable and all the visual-spatial measures (MAP, SRT, STICK, VPT2, VPT4, and VMI) as independent variable. For each analysis, the statistical criterion for entry was a probability of *p* ≤ 0.05, with the criterion for subsequent removal probability of *p* ≥ 0.1. A *p*-value less than 0.05 was considered as statistically significant.

## Results

### Differences in Visual-Spatial Abilities between DD and NR According to Educational Stages

Results of the MANOVA (Group × Task × Subgroup) showed a significant effect of Group (*F*_(1,121)_ = 25.31, *p* < 0.00001), with higher scores for NR than for children with DD, a significant effect of Task (*F*_(5,605)_ = 28.63, *p* < 0.0001) and a significant effect of Subgroup (*F*_(1,121)_ = 10.87, *p* = 0.001). The Group × Task effect and the Group × Subgroup effect were found non-significant (respectively, *F*_(5,605)_ = 1.98, *p* = 0.08; *F*_(1,121)_ = 0.68, *p* = 0.41), while the effect Subgroup × Task resulted statistically significant (*F*_(5,605)_ = 7.66, *p* < 0.00001). A significant effect Group × Task × Subgroup was also found (*F*_(5,605)_ = 3.01, *p* = 0.01).

*Post hoc* analysis revealed that the younger subgroup of children with DD performed significantly worse than younger NR in SRT (*p* = 0.0032), in both visual perception tasks (VPT2 *p* = 0.040 and VPT4 *p* = 0.017) and in VMI (*p* = 0.000004). No significant difference was found between younger children with DD and NR in MAP (*p* = 0.86) and STICK (*p* = 0.08).

However, older subgroup of children with DD performed significantly worse than older NR in MAP (*p* = 0.002), SRT (*p* = 0.029), and VPT4 (*p* = 0.0022). No significant difference was found between older children with DD and NR in STICK (*p* = 0.64), VPT2 (*p* = 0.17), and VMI (*p* = 0.07).

**FIGURE 1:**
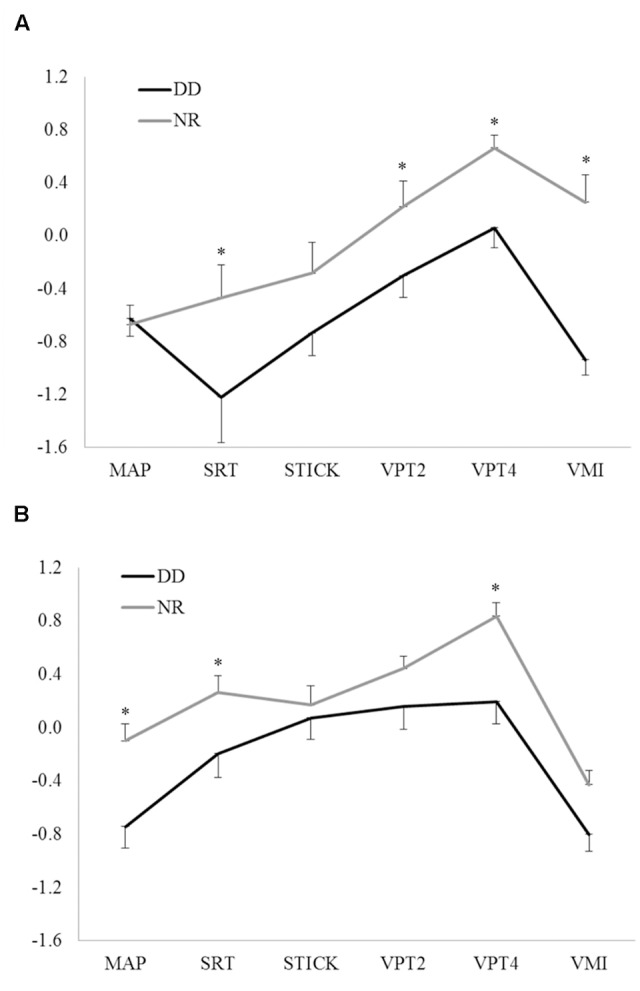
**Effect Group × Task × Subgroup of the MANOVA with means and standard errors of each visual-spatial measure in younger (A)** and older **(B)** subgroups of children. DD, Developmental Dyslexia; NR, Normal Readers; MAP, Map Mission; SRT, Spatial Rotation Test; STICK, Stick Test; VPT2, Visual Perception Test-subtest 2; VPT4, Visual Perception Test-subtest 4; VMI, Visual Motor Integration Task. ^∗^ indicates *p* < 0.05.

**Figure 1** showed the effect Group × Task × Subgroup and Panel A reports means and standard errors of each visual-spatial task in younger subgroups of children with DD and NR, while Panel B reports those of each visual-spatial task in older subgroups of children with DD and NR.

*Post hoc* analysis revealed that older children with DD performed significantly better than younger children with DD in SRT (*p* = 0.00001), VPT2 (*p* = 0.04), and STICK (*p* = 0.0005), while no differences have been detected between younger children with DD and older children with DD in VPT4 (*p* = 0.56), VMI (*p* = 0.056), MAP (*p* = 0.61). Conversely, older NR scored significantly higher than younger NR in SRT (*p* = 0.002) and MAP (*p* = 0.01). In VMI, older NR performed significantly worse than younger NR (*p* = 0.004). No differences were found between older NR and younger NR in VPT4 (*p* = 0.46), VPT2 (*p* = 0.34), and STICK (*p* = 0.06).

### Predictors of Reading Abilities in Younger and Older Participants

**FIGURE 2:**
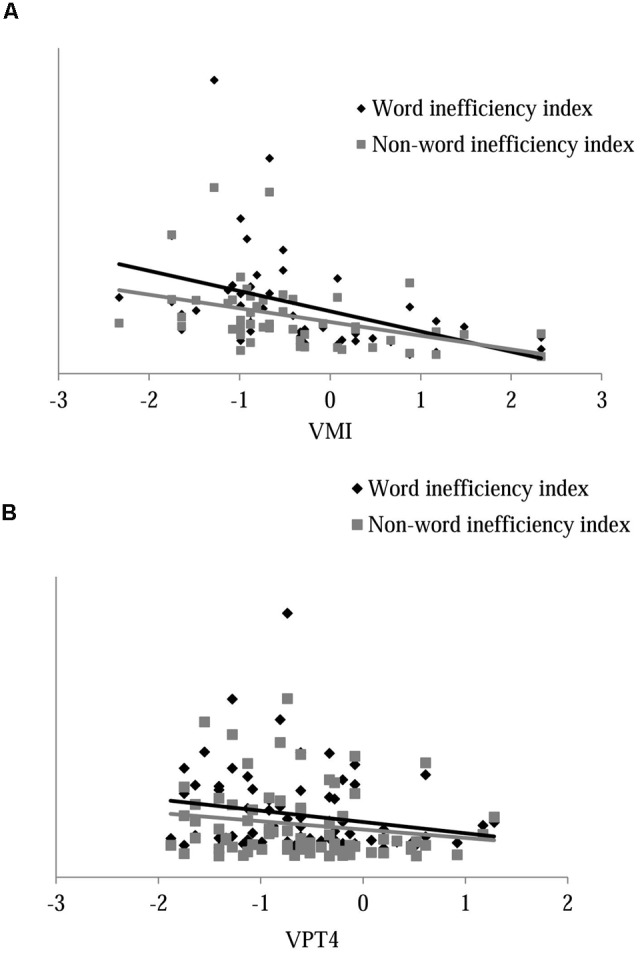
**Regression graph for word and non-word inefficiency index in younger (A)** and older **(B)** subgroups of children. VPT4, Visual Perception Test-subtest 4; VMI, Visual Motor Integration Task.

In order to verify whether visual-spatial measures could be potential predictors of reading abilities, a regression analysis was separately performed for younger and older children. A stepwise method, in which the word and non-word inefficiency index was entered as the dependent variable and visual-spatial measures as the independent variables, was applied. In younger participants, results showed that VMI significantly predict word and non-word inefficiency reading index accounted, respectively, for 16.3% (*F*_(1,48)_ = 9.36, *p* = 0.004) and for 15.0% (*F*_(1,48)_ = 8.44, *p* = 0.006) of the variance. **Figure 2A** illustrates the relation between word and non-word inefficiency index and VMI in younger children. In older participants, VPT4 was found as a significant predictor of word and non-word inefficiency reading index. In detail, VPT4 accounted for the 14.6% of the variance (*F*_(1,73)_ = 12.43, *p* = 0.001) of word inefficiency reading index and for the 12.5% (*F*_(1,73)_ = 10.46, *p* = 0.002) of non-word inefficiency reading index. **Figure 2B** illustrates the relation between word and non-word inefficiency index and VPT4 in older children. **Table 3** illustrates detailed results of the regression analysis.

**Table 3:** Results of stepwise regression analyses in younger and older participants.

	Word Inefficiency Index			Non-word Inefficiency Index		
	β	*t*	*p*	β	*t*	*p*
**Younger**						
MAP	–0.027	–0.197	0.844	–0.006	–0.043	0.966
SRT	–0.032	–0.232	0.818	0.018	0.131	0.896
STICK	–0.009	0.060	0.953	–0.015	–0.104	0.918
VPT2	–0.077	–0.538	0.593	–0.066	–0.457	0.650
VPT4	–0.103	–0.766	0.448	–0.074	–0.541	0.591
VMI	–0.404	–3.060	0.004^∗^	–0.387	–2.907	0.006^∗^
**Older**						
MAP	–0.209	–1.881	0.064	–0.171	–1.508	0.136
SRT	–0.047	–0.401	0.689	–0.061	–0.518	0.606
STICK	0.027	0.242	0.809	0.026	0.235	0.815
VPT2	0.033	0.282	0.779	0.052	0.443	0.659
VPT4	–0.381	–3.526	0.001^∗^	–0.354	–3.235	0.002^∗^
VMI	–0.137	–1.247	0.216	–0.110	–0.985	0.328

*MAP, Map Mission; SRT, Spatial Rotation Test; STICK, Stick Test; VPT2, Visual Perception Test-subtest 2; VPT4, Visual Perception Test-subtest 4; VMI, Visual Motor Integration Task. ^∗^ indicates *p* < 0.01.*

## Discussion

The main aim of the present study was to investigate differences in visual-spatial abilities in children with DD compared to age-matched NR in two different educational stages. Regression analyses were also performed to verify whether different visual-spatial abilities are involved in reading process according to different educational stages.

Results revealed that younger children with DD performed significantly worse than NR in a mental rotation task (SRT), a more-local visual-spatial task (VPT2), a more-global visual-perceptual task (VPT4) and a visual-motor integration task (VMI). Our findings are similar to those found in the study by Rüsseler et al. (2005), where younger children with DD, compared to NR, were impaired in solving three mental rotation tasks and the Embedded Figures Test, a test assessing the ability to detect hidden figures in complex patterns comparable to our VPT4. However, in another study (Del Giudice et al., 2000) investigating visual-spatial cognition and memory in 43 children (aged 8–9 years) with reading impairments, participants with DD did not differ from children that received a diagnosis of DD and then recovered reading deficits at 1-year follow-up (control group) in the visual-spatial task adopted. Since children without any history of reading disability were included as a control group in our study, we clearly differentiated visual-spatial processes of children with DD from those of NR with respect to the study by Del Giudice et al. (2000), in which the control group comprised children had recovered reading deficits. Therefore, we believe our results are more informative regarding the contribution of visual-spatial abilities in DD.

Moreover, our results documented deficits in several visual-spatial abilities in the younger subgroup with DD, as shown by different tasks (i.e., SRT, VPT2, VPT4, and VMI), that could contribute to negatively affect reading skills in children with DD at the first educational stage. Many studies investigated the relationship between VMI and the quality of handwriting (see, for example, Karlsdottir and Stefansson, 2002; Kaiser et al., 2009). Since dysgraphia is known to be associated with DD, low scores in VMI in our younger participants might reflect poorer skills and/or less experience in handwriting. Further studies are needed to better investigate the relationship between visual-motor integration difficulties, poor handwriting and reading disorders at different educational stages. However, deficits found in VPT2 and in VMI were not documented in the older subgroup with DD, which, in turn, showed deficits in the more-global visual-perceptual task (VPT4), in the mental rotation task (SRT) and in the visual attention task (MAP). *Post hoc* comparison between younger and older children with DD showed that the younger subgroup with DD obtained significantly lower scores than the older subgroup in VPT2, STICK and SRT while no difference emerged in VPT4, MAP, and VMI.

Results from previous studies using rotation tasks in children with DD aged, similarly to our older subgroup, between 11–13 years (Corballis et al., 1985) and 10–12 years (Wang and Yang, 2011) failed to find differences between DD and NR. A possible explanation for this discrepant result from our study could be found in the characteristics of the tasks to evaluate mental rotation. Indeed, when the rotation tasks were presented as computer games, no deficits were found in participants with DD (Corballis et al., 1985; Wang and Yang, 2011) while when it was used a paper and pencil task, more similar to the one adopted in our study, a deficit in mental rotation abilities was found in students with DD (Winner et al., 2001). Concerning the present findings on visual attention in older children with DD, deficits have been repetitively described in literature using psychophysical experiments. Specifically, results evidenced in DD reduced visual-attention span in task requiring to process multiple elements in parallel (Bosse and Valdois, 2003; Bosse et al., 2007; Bosse and Valdois, 2009; Lobier et al., 2014; Lobier and Valdois, 2015). This visual processing deficit has been interpreted as strictly connected to the reading impairment due to the limitation of the ability of the visual-attention window to spread over a whole word, and then to identify words with fast and parallel procedures. As regards to VMI task, in the older subgroup with DD, our results are consistent with those reported by Goldstand et al. (2005), that failed to find differences in visual processing between NR and children with DD.

The regression analyses of our study documented that in younger participants, independently of the group (children with DD or NR), VMI significantly predicted word and non-word inefficiency reading index. However, in older participants, the only significant predictor of word and non-word inefficiency reading index was VPT4. VMI is a task designed to investigate visual perceptive abilities and the ability to use visual information to guide motor behavior, referred to as visual-motor integration, and it substantially includes a wide range of abilities as visual-spatial perceptive abilities, fine motor abilities and motor planning. The visual-spatial perceptive abilities required by VMI include both the analysis of the spatial location, orientation and the visual-object recognition to perceive the global form of the figure. A possible interpretation of the regression results concerning VMI measures and reading deficits is that during the first educational stage, more complex and extensive visual-spatial abilities could be required for reading. Neuroanatomically, when children are in the first educational stage, there is a strict connection between the dorsal and ventral stream, and the angular and supramarginal gyri seem to help the ventral regions to focus on individual letters in order to identify them and their order (Stein, 2014).

On the other hand, in our older children only the visual-perceptual task VPT4 significantly predicted reading measures. To solve VPT4, the form recognition of the figure is required, regardless of changes in the surrounding environment and the primary involvement of the ventral stream is expected (Hebart and Hesselmann, 2012). Even if speculatively, we could hypothesize that in this later educational stage the contribution of visual-spatial abilities to reading relates to a more global perception strategy to analyze the shape of the word. Indeed, as the children grow-up, they become more expert in reading and apply a whole recognition strategy to identify a word. Accordingly, the ventral word form area (VWFA), located in the fusiform gyrus, seemed to play an important role in whole word recognition and in the form analysis of the words (Stein, 2014), as identified by a number of neuroimaging studies (Cohen et al., 2000, 2002; Dehaene and Cohen, 2011). When the more expert-reader has improved the lexicon, the VWFA rapidly recognizes the whole strings and allocates to it the meaning (Stein, 2014).

Our results could contribute to clarify the relationship between reading and a number of visual-spatial abilities at different educational stages in DD, indicating that in the first educational stage more complex and extensive visual-spatial deficits could interfere in exploring letters and words while in the next educational stage more-global perceptual deficits could hinder the reading process of children with DD.

Studies failing to find visual-spatial deficits in children or adults with DD did not include children at different educational stages and tended to investigate only single aspects of the visual-spatial domain (Corballis et al., 1985; Del Giudice et al., 2000; White et al., 2006; Wang and Yang, 2011). However, our results stress the importance of considering different visual-spatial domains and different educational stages to better understanding the relationship between reading and visual-spatial abilities in DD.

A limitation of the study is that only cross-sectional comparisons were performed. In order to examine the change of the relationship between visual-spatial abilities and reading acquisition in DD a more developmental study design, including either cross sectional analysis or longitudinal data, should be developed in future. Moreover, caution should be taken in generalizing our results to other languages with a reduced orthographic-phonological correspondence. Indeed, in languages with less transparent orthography, reading processes could require a different contribution of local and global visual-spatial abilities at different educational stages. Further studies are needed in order to extend present results to other languages.

Reading is a complex cognitive process, in which not only phonological skills, but memory, attention, automatization and visual-spatial skills are involved. The present study focused on the contribution of visual-spatial abilities at different educational stages in affecting reading. Further studies are needed in order to consider the role of the different underlying neurocognitive deficits in DD at different developmental stages.

## Author Contributions

DM, GG, and ST developed the study concept and all authors designed the study. GG, ST performed the data collection and the data analysis under the supervision of DM and SV. GG and ST drafted the paper and DM and SV provided critical interpretation of the results and revisions. All authors read and approved the final version to be submitted.

## Conflict of Interest Statement

The authors declare that the research was conducted in the absence of any commercial or financial relationships that could be construed as a potential conflict of interest.
